# Characterization of the complete mitogenome of brown-spotted locust, *Cyrtacanthacris tatarica* (Orthoptera: Acrididae: Cyrtacanthacridinae)

**DOI:** 10.1080/23802359.2022.2059409

**Published:** 2022-04-04

**Authors:** Ran Li, Lina Jiang, Yujian Li

**Affiliations:** School of Life Sciences, Qufu Normal University, Qufu, Shandong, PR China

**Keywords:** Mitochondrial genome, phylogeny, Orthoptera, Cyrtacanthacridinae, *Cyrtacanthacris tatarica*

## Abstract

The complete mitochondrial genome of *Cyrtacanthacris tatarica* was firstly sequenced and analyzed. The circular mitogenome was 15,679 bp long, showing a bias of AT rich on the majority strand (42.34% of A, 29.99% of T, 11.19% of G, and 16.18% of C). It consisted of the typical 37 genes (13 protein-coding genes, two ribosomal RNA genes, and 22 transfer tRNA genes) and one longest non-coding region (called as control region). All PCGs used standard ATN initiation codons, and most PCGs were terminated with complete codons (TAA/TAG), apart from *cox1* and *nad5*. Phylogenetic analyses based on the concatenated nucleotide sequences of PCGs supported that Cyrtacanthacridinae was monophyletic, and the sister group relationship between *C. tatarica* and *Schistocerca gregaria gregaria* was determined. Our results may provide molecular information for the genetic evolution and taxonomy of the acridid species.

Mitochondrial genomes (mitogenomes) are being increasingly employed to explore the evolution and phylogenetic relationships in diverse insect taxa (Shi et al. 2019; Yang et al. 2020). *Cyrtacanthacris tatarica* (Linnaeus, 1758) belongs to the genus *Cyrtacanthacris* (Orthoptera: Cyrtacanthacridinae), which is considered as a potential pest for the scattered vegetation of herbs and shrubs (Elsayed et al. 2014). To data, no species within the genus is known for the mitogenome sequence. In order to better understand the characteristics of the mitogenome of *Cyrtacanthacris* and explorer its phylogenetic status, we determined and annotated the whole sequence of *C. tatarica* for the first time.

In this study, the mitogenome of *C. tatarica* was sequenced using the specimen captured from Sanya in Hainan Province, China (18°14′24″N, 109°36′49″E). The samples were immediately deposited in 100% ethanol and stored at −20 °C until processing. The entire genome was extracted from the femoral muscle using a Wizard^®^ Genomic DNA Purification Kit (Promega, Madison, WI). The experimental sample and DNA were preserved in the laboratory of School of Life Sciences, Qufu Normal University, China (specimen voucher number LYJ2021CT; corresponding author: Yujian Li is in charge of the deposited sample). PCR amplification was performed with several universal primer pairs for grasshoppers (Simon et al. 2006). The obtained sequenced fragments were assembled to the final complete genome through the program SeqMan (Burland 2000). Sequence annotation was performed using MITOS Web Server and then manually checked according to its relative species (Bernt et al. 2013). The specimens were collected from meadow; no specific permissions were required for the locations. The species in our study is agricultural pest and is not included in the ‘List of Protected Animals in China’.

The gene composition and order of this circular mitogenome was identical with those of other acridids reported before (Li et al. 2020). It encoded for 13 protein-coding genes (PCGs), 22 transfer RNA genes (tRNAs), two ribosomal RNA genes (rRNAs) as well as one control region (GenBank accession number: MK352102). The sequence was 15,679 long, showing a bias of AT rich on the majority strand (42.34% of A, 29.99% of T, 11.19% of G, and 16.18% of C). All PCGs started with the conventional initiation codon ATN (eight with ATG). Most genes were terminated with the complete stop codons (TAA and TAG), while the incomplete termination codon (T) was identified in two genes *cox1* and *nad5*. Two rRNA genes (*rrnL* and *rrnS*) were 1315 bp and 796 bp in size, respectively. All 22 typical tRNA genes were identified ranging from 63 to 73 bp long in the generated mitogenome. In addition, the control region was located in the conserved position between the genes *rrnS* and *trnV* with the size of 772 bp. There were 12 intergenic spacers varying from 1 bp to 20 bp in size, and the largest region was present between *trnL2* and *cox2*. Besides, eight gene overlap regions from 1 bp to 8 bp were found in this mitogenome. In addition, the nucleotide sequence ‘ATGATAA’ between *atp6* and *atp8* was identical to that of other acridid mitogenomes, which is a common feature among species in Acrididae.

The dataset of all PCGs with three codon positions was used in the present phylogenetic analyses. The phylogenetic trees generated by MrBayes 3.2.7a (Bayesian Inference) and RAxML 8.2.0 (maximum likelihood) yielded the same topologies (Ronquist et al. 2012; Stamatakis 2014). As shown in Figure 1, C. *tatarica* was clustered with other species from Cyrtacanthacridinae, and our analyses supported the subfamily was a monophyletic group. Additionally, *C. tatarica* was demonstrated to be the sister group with *Schistocerca gregaria gregaria*. The results will lay a foundation for the evolutionary and phylogenetic study of Cyrtacanthacridinae as well as Acrididae.

**Figure 1.**
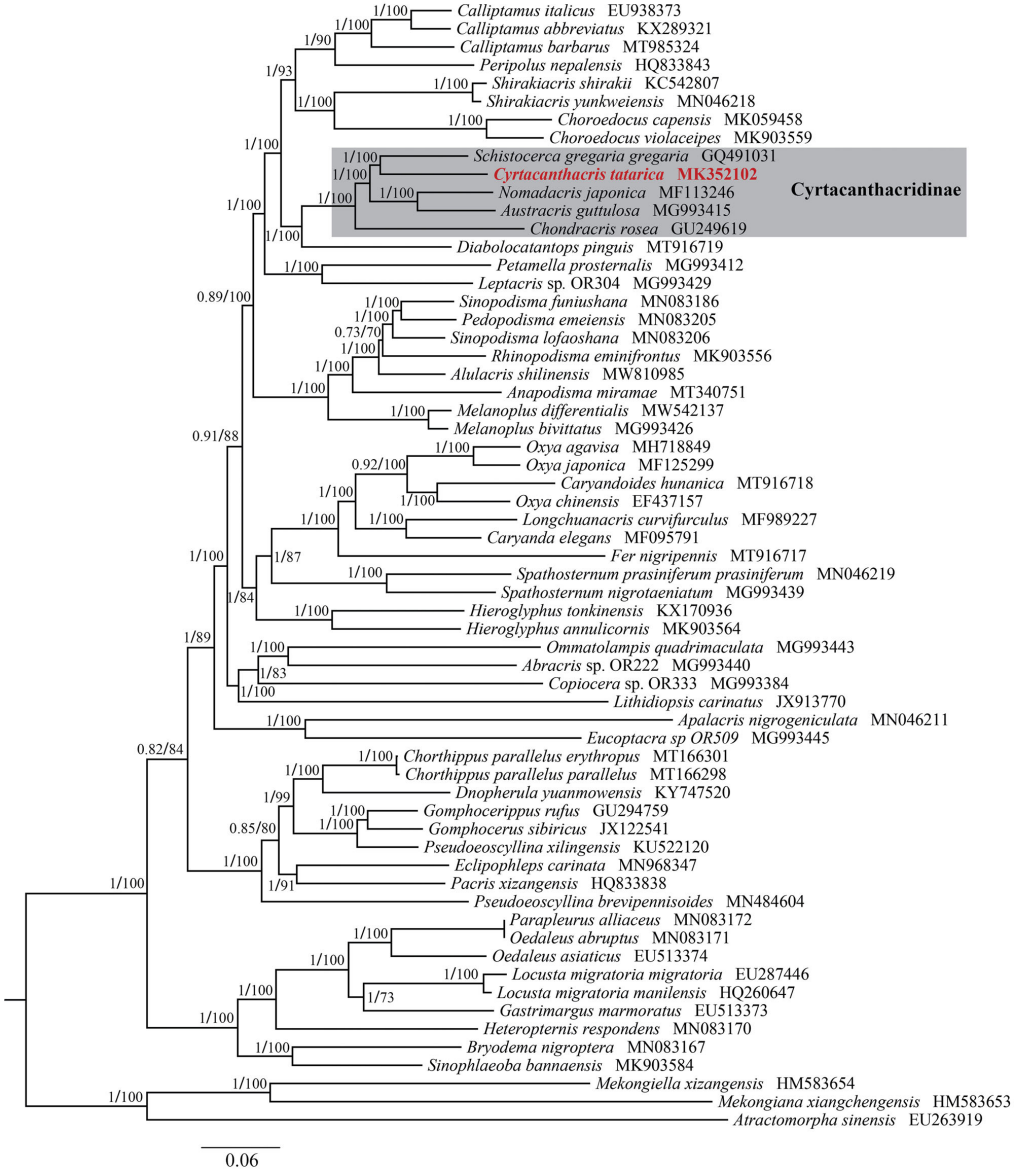
Phylogenetic tree obtained from ML and BI analysis based on 13 concatenated mitochondrial PCGs. Numbers on node are posterior probability (PP) and bootstrap value (BV).

## Authors contributions

Ran Li conceived and designed the experiments, performed the experiments, analyzed the data, prepared figures and/or tables, authored or reviewed drafts of the paper, and approved the final draft. Lina Jiang performed the experiments, prepared figures and/or tables, and approved the final draft. Yujian Li conceived and designed the experiments, analyzed the data, authored or reviewed drafts of the paper, and approved the final draft. All authors agree to be accountable for all aspects of the work.

## Data Availability

The genome sequence data that support the findings of this study are openly available in GenBank of NCBI at https://www.ncbi.nlm.nih.gov under the accession no. MK352102. Our mitogenome sequence was generated by Sanger sequencing in this study, so there is no BioProject, SRA, and BioSample accession numbers, and the genome sequence data have been released in GenBank of NCBI.
